# Supplementary figures and images for: Chromatin accessibility established by Pou5f3, Sox19b and Nanog primes genes for activity during zebrafish genome activation
Source: PLoS Genet. 2020 Jan 15;16(1):e1008546. doi: 10.1371/journal.pgen.1008546 (PMC6986763; doi:10.1371/journal.pgen.1008546)

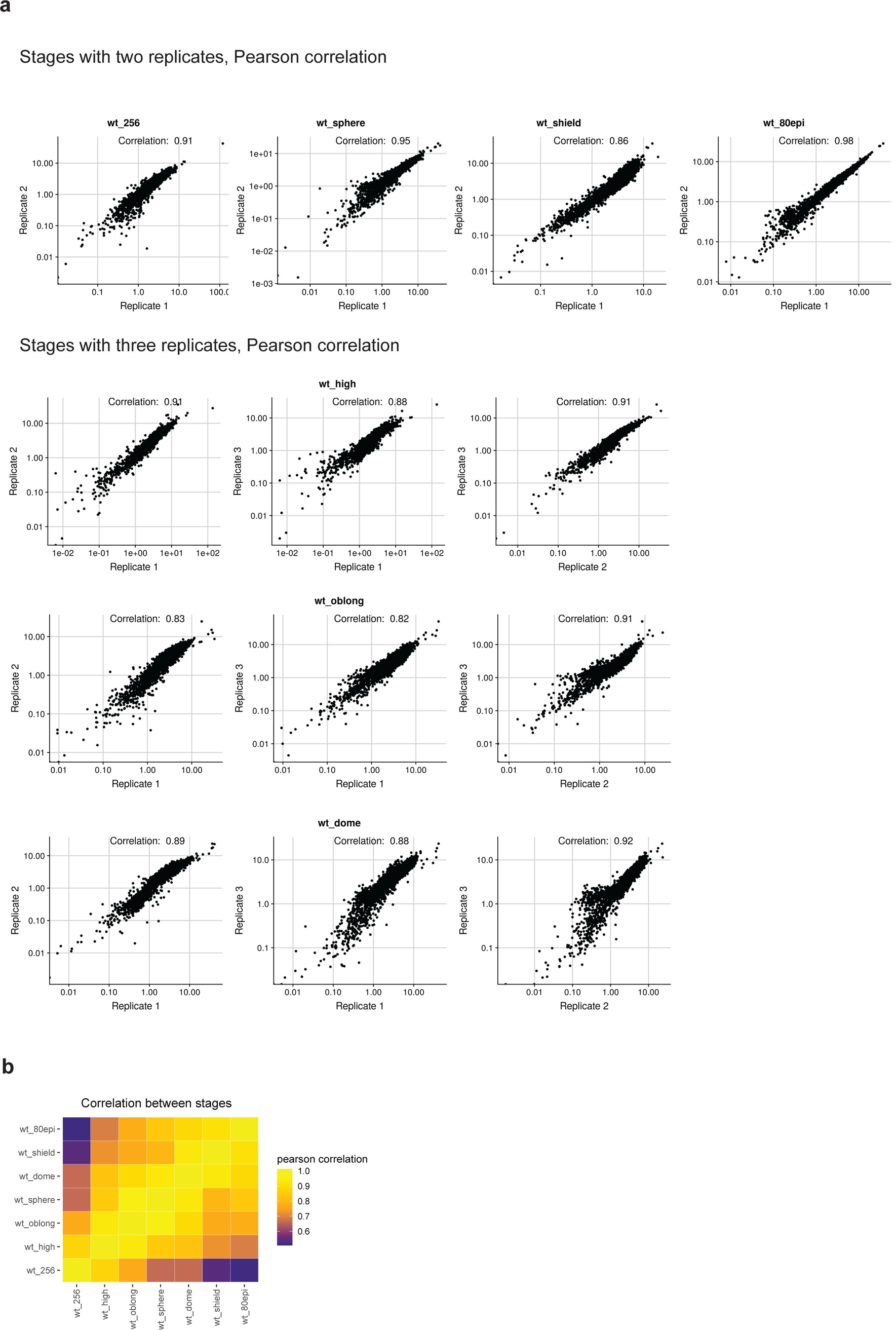

Supplement: S1 Fig — a) Pearson correlation between individual ATAC-seq replicates. Two biological replicates were generated for 256-cell, sphere, shield and 80% epiboly stage, and three biological replicates for high, oblong and dome stage. b) Pearson correlation between pooled replicates of different stages. (TIF) [file pgen.1008546.s001.tif]

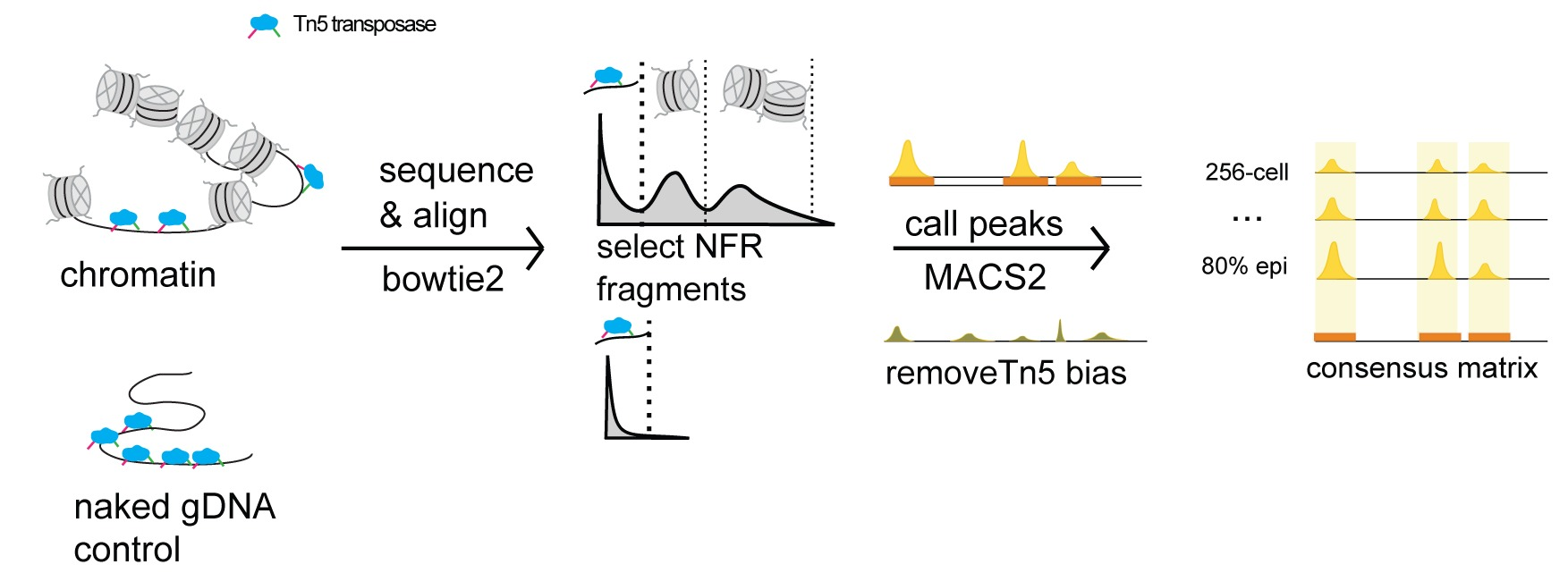

Supplement: S2 Fig — Chromatin and naked genomic DNA from zebrafish embryos was tagmented using Tn5 transposase, followed by sequencing and alignment to the genome (bowtie2). Fragments of sub-nucleosomal size (nucleosome free regions, NFR) were selected by applying a 130bp cut-off after visual inspection of the fragment length profiles. Peaks were called using MACS2 using the signal obtained from the naked DNA sample as background. Peaks called in each wild-type stage were merged across the time-course to generate a consensus peak set for further analysis. (TIF) [file pgen.1008546.s002.tif]

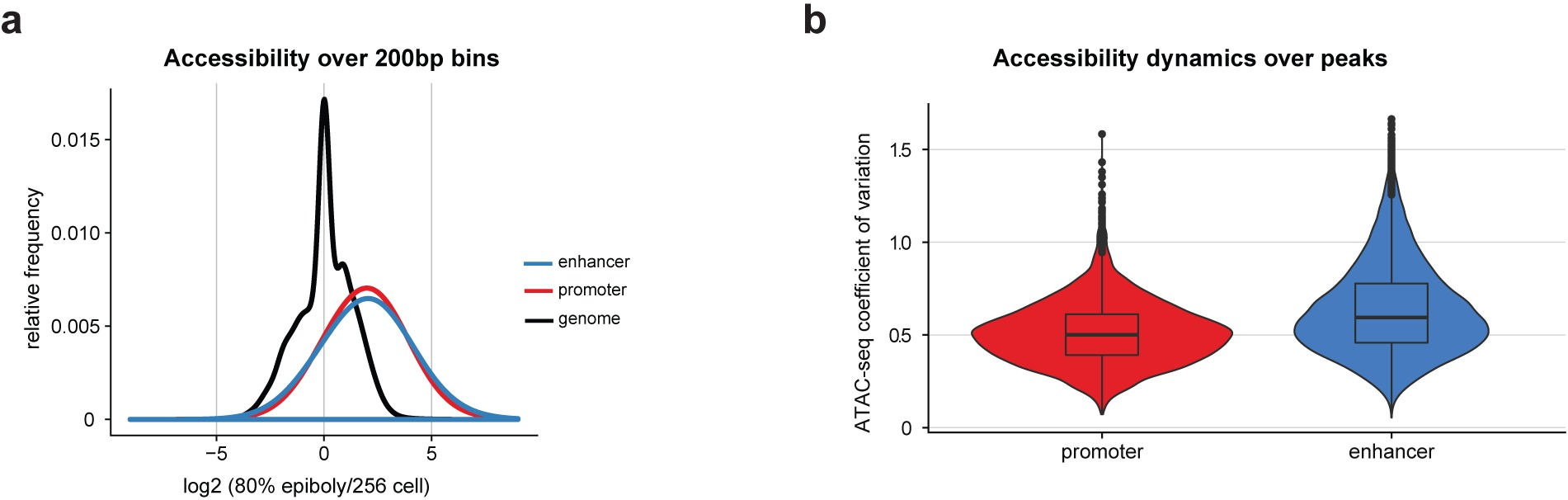

Supplement: S3 Fig — a) Average accessibility increase across the time-series (from 256-cell to 80% epiboly) over 200bp bins genome-wide (black), at promoters (red), and putative enhancer elements (blue). b) The coefficient of variation of normalized accessibility across the time course for promoter and putative enhancer associated peaks. A higher value indicates more variation in accessibility across stages. (TIF) [file pgen.1008546.s003.tif]

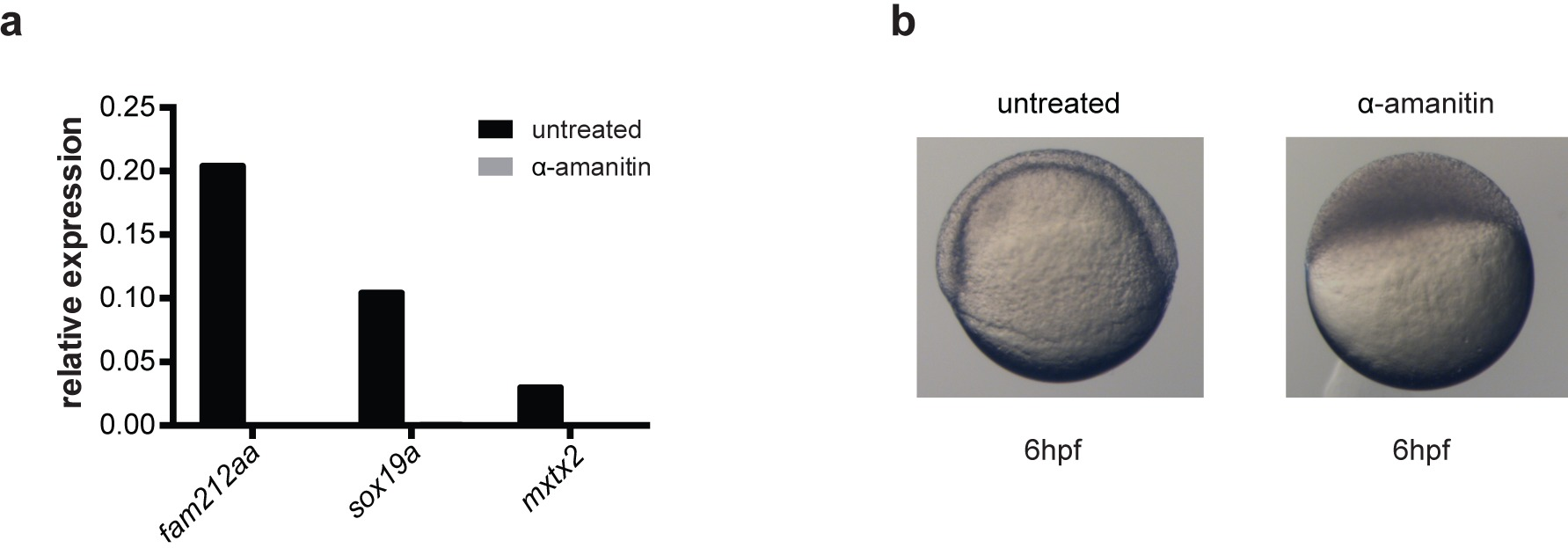

Supplement: S4 Fig — a) Gene expression levels of the zygotically activated genes fam212aa, sox19a and mxtx2 in untreated and α-amanitin-treated embryos. α-amanitin treatment results in transcription inhibition. b) Brightfield images of zebrafish embryos at 6hpf in untreated and α-amanitin-treated embryos. α-amanitin treatment results in embryonic arrest at sphere stage. (TIF) [file pgen.1008546.s004.tif]

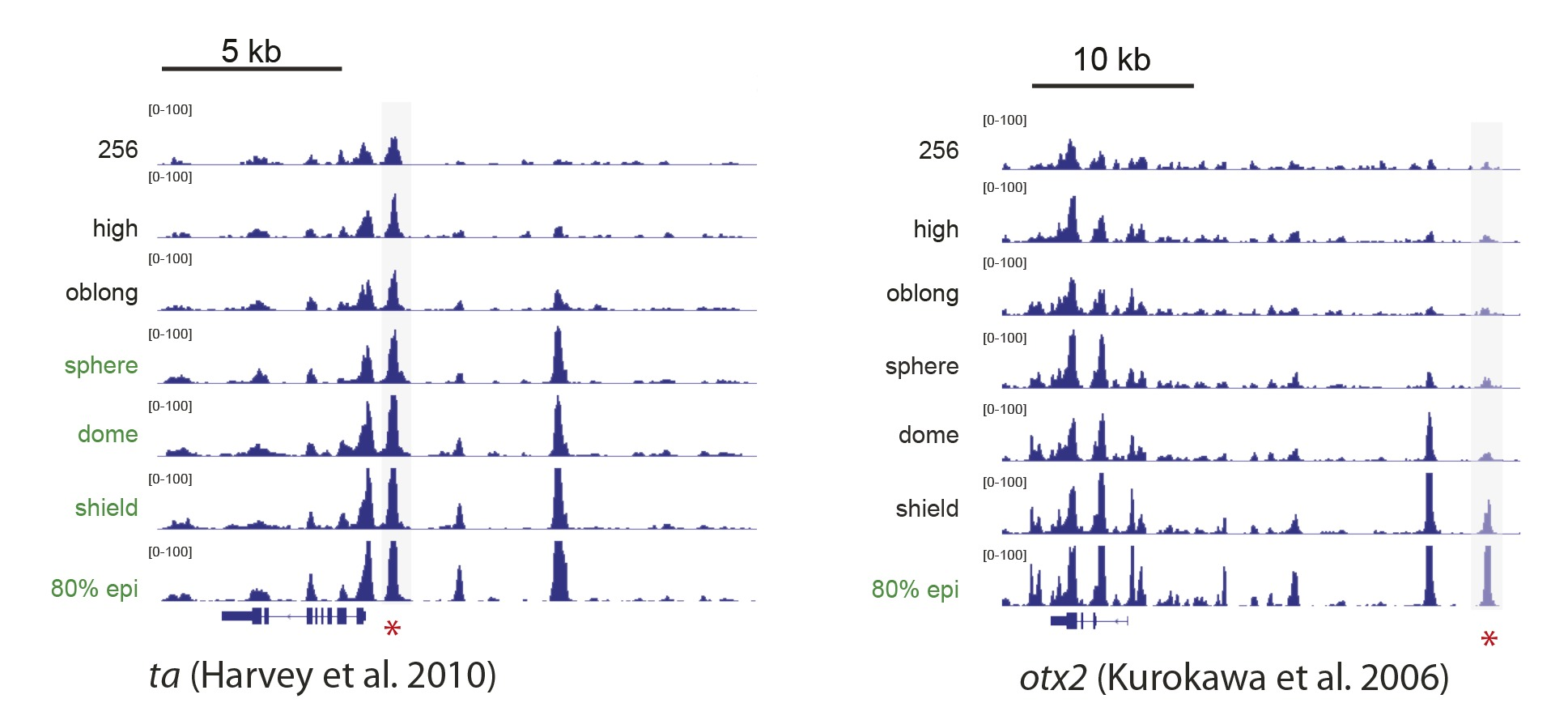

Supplement: S5 Fig — Genome browser snapshots showing the ta and otx2 gene loci. Green fonts indicate the stages when the gene is active. Experimentally verified enhancers for these genes [38,39] are marked by a red asterisk. (TIF) [file pgen.1008546.s005.tif]

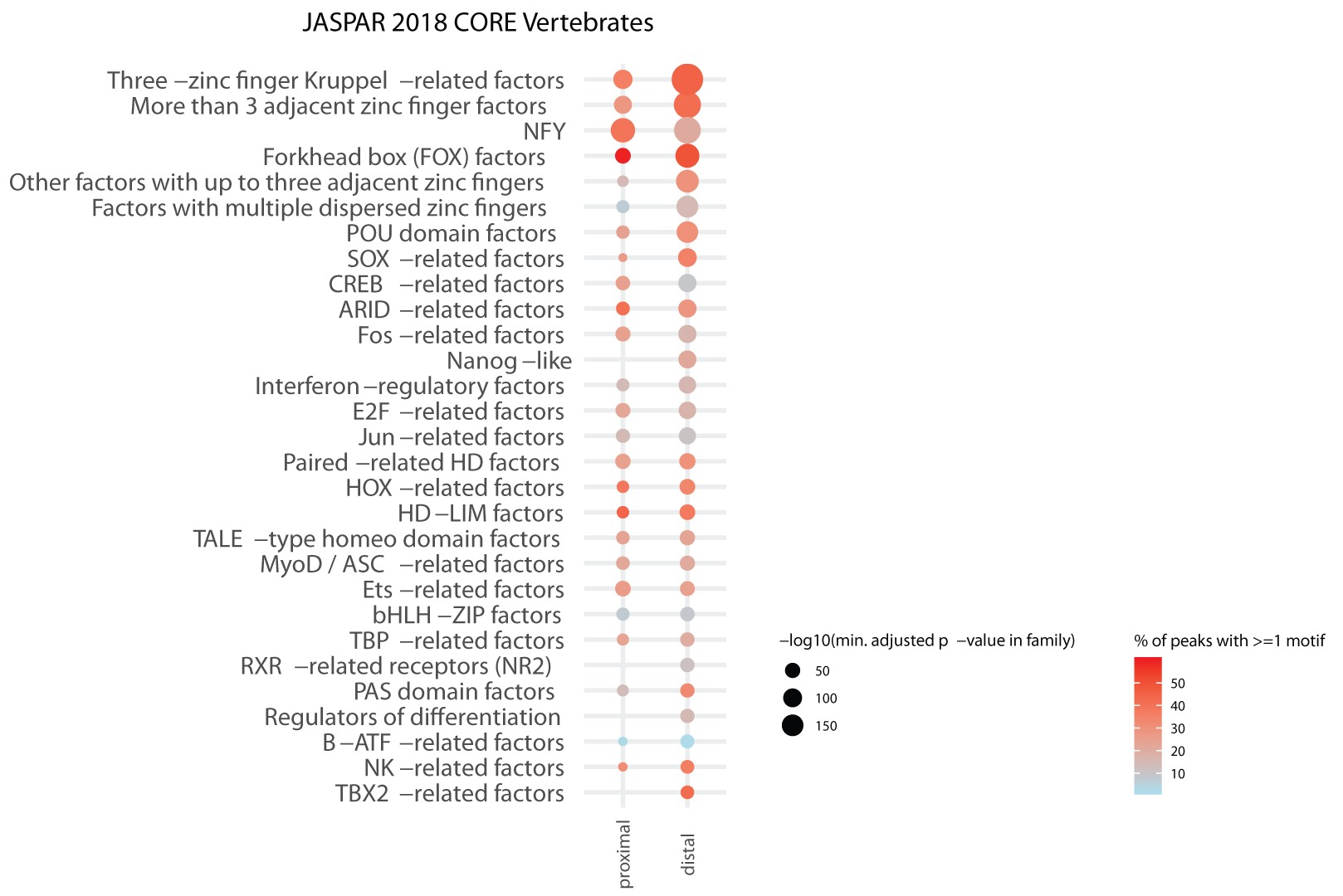

Supplement: S6 Fig — Enrichment of TF motifs from the JASPAR vertebrates database (with the custom Nanog motif from [24]) at proximal (within +/- 1kb of TSS) and distal (enhancer/intergenic) regions that show an increase in accessibility between 256-cell and oblong stage. Individual motifs were summarized at the motif-family level and the minimum adjusted p-value for each family is indicated by the circle size in the plot. The color scale ranging from blue (low) to red (high) indicates the % of peaks with at least one motif from the respective motif-family. (TIF) [file pgen.1008546.s006.tif]

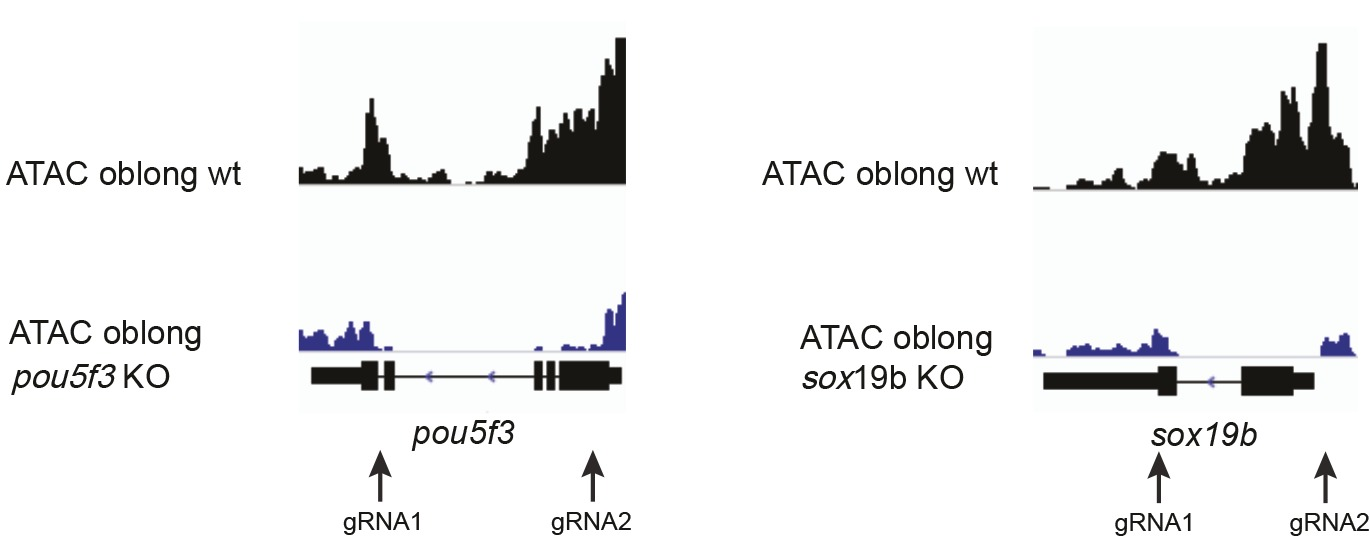

Supplement: S7 Fig — Genome browser snapshots showing ATAC accessibility tracks at pou5f3 and sox19b in wild-type and knock-out embryos. The absence of reads over the gene body of pou5f3 and sox19b in knock-outs confirms the deletion of the loci. Location of the gRNAs used for deleting the genes are indicated with arrows. (TIF) [file pgen.1008546.s007.tif]

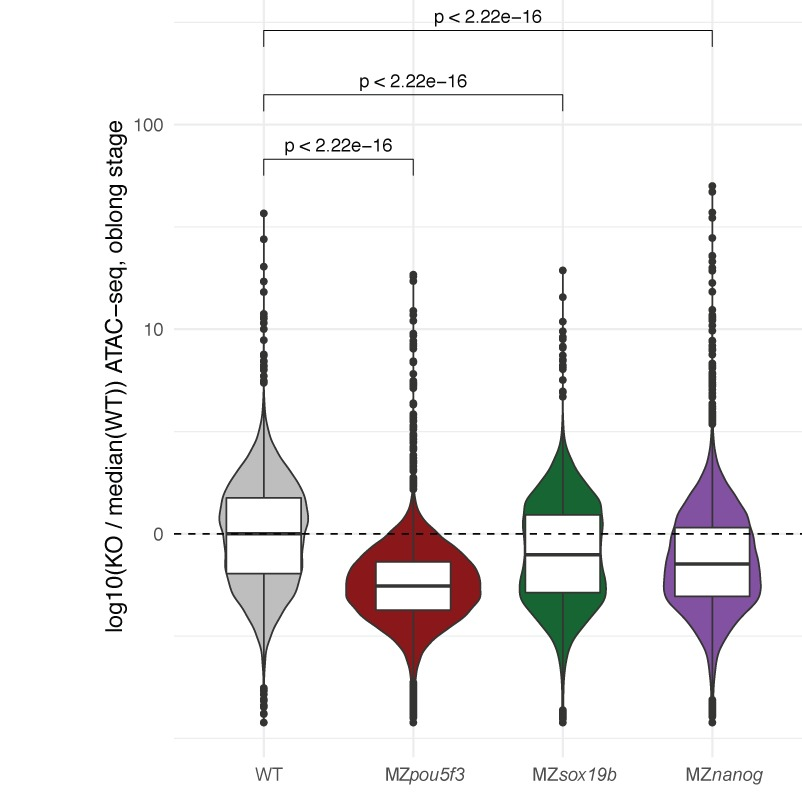

Supplement: S8 Fig — Violin plots show the aggregated fold change in accessibility in MZpou5f3, MZsox19b and MZnanog mutants compared to wild-type embryos at oblong stage. Significance of differences was tested using paired, one-sided, t-tests. (TIF) [file pgen.1008546.s008.tif]

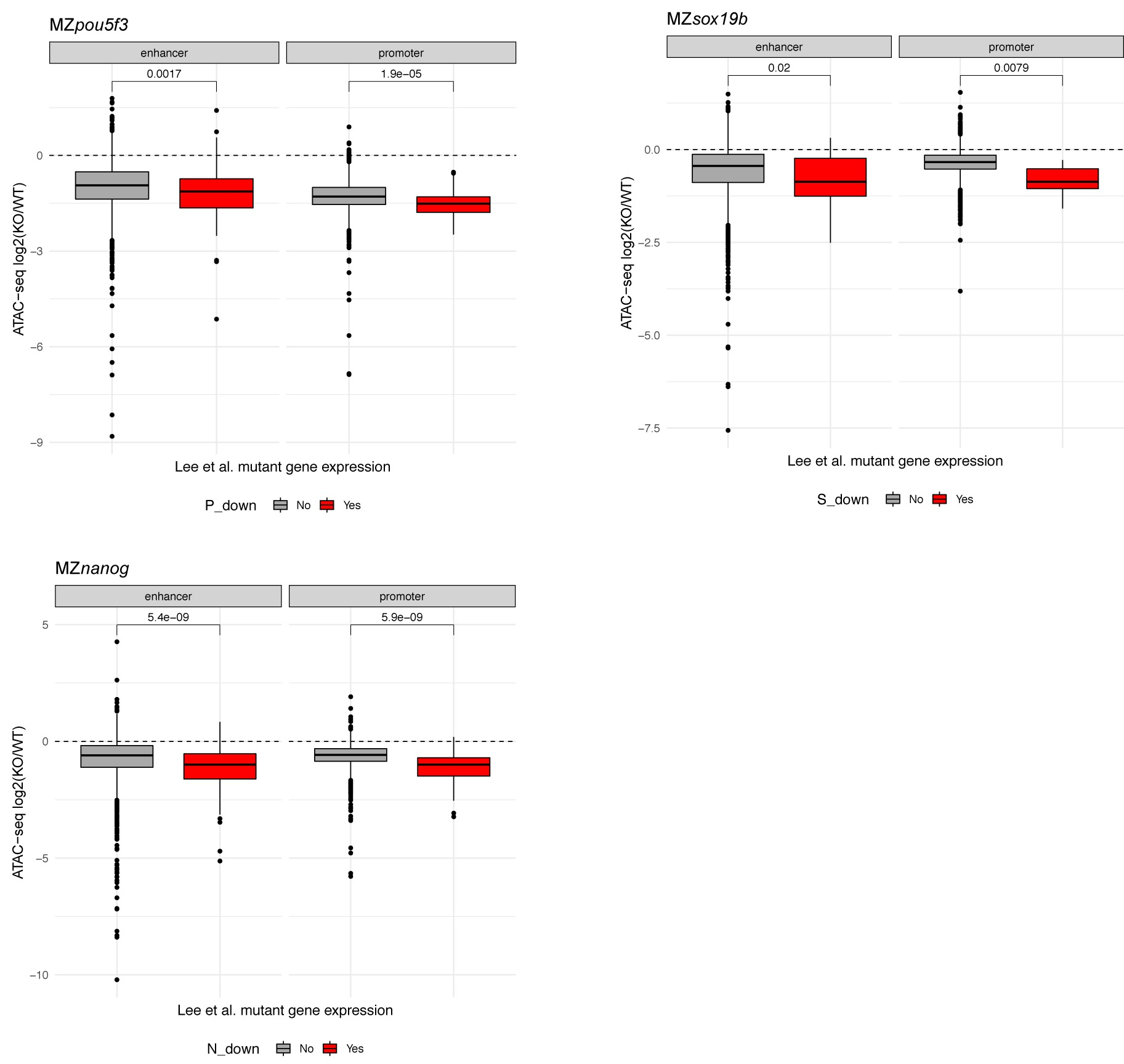

Supplement: S9 Fig — Boxplots show the log2 fold change in accessibility in mutants compared to wild-type embryos (oblong stage) at putative enhancers and promoters associated with genes that are downregulated upon Pou5f3, Sox19b and Nanog knock-down (red) and genes that are not downregulated (grey). Expression data is from [15]. Statistical significance of the differences was tested by two-sample Welch t-tests. (TIF) [file pgen.1008546.s009.tif]

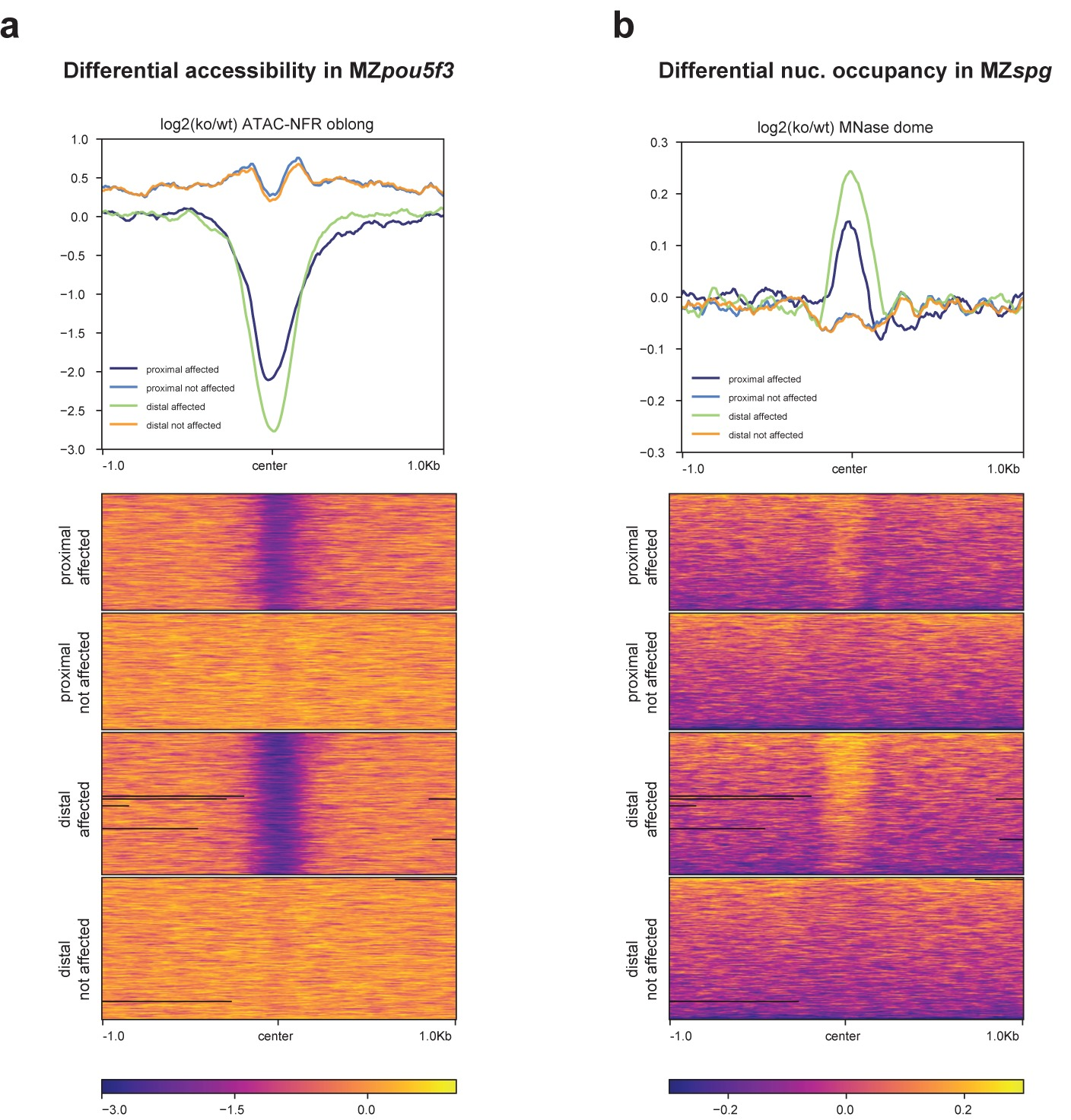

Supplement: S10 Fig — a) Metagene profiles and heatmaps showing the log2 fold change in accessibility in MZpou5f3 mutants compared to wild-type at oblong stage. b) Metagene profile and heatmaps for the same regions as in a), showing the log2 fold change in nucleosome occupancy in pou5f3 (MZspg) mutants compared to wild-type at dome stage, using data from [27] (TIF) [file pgen.1008546.s010.tif]

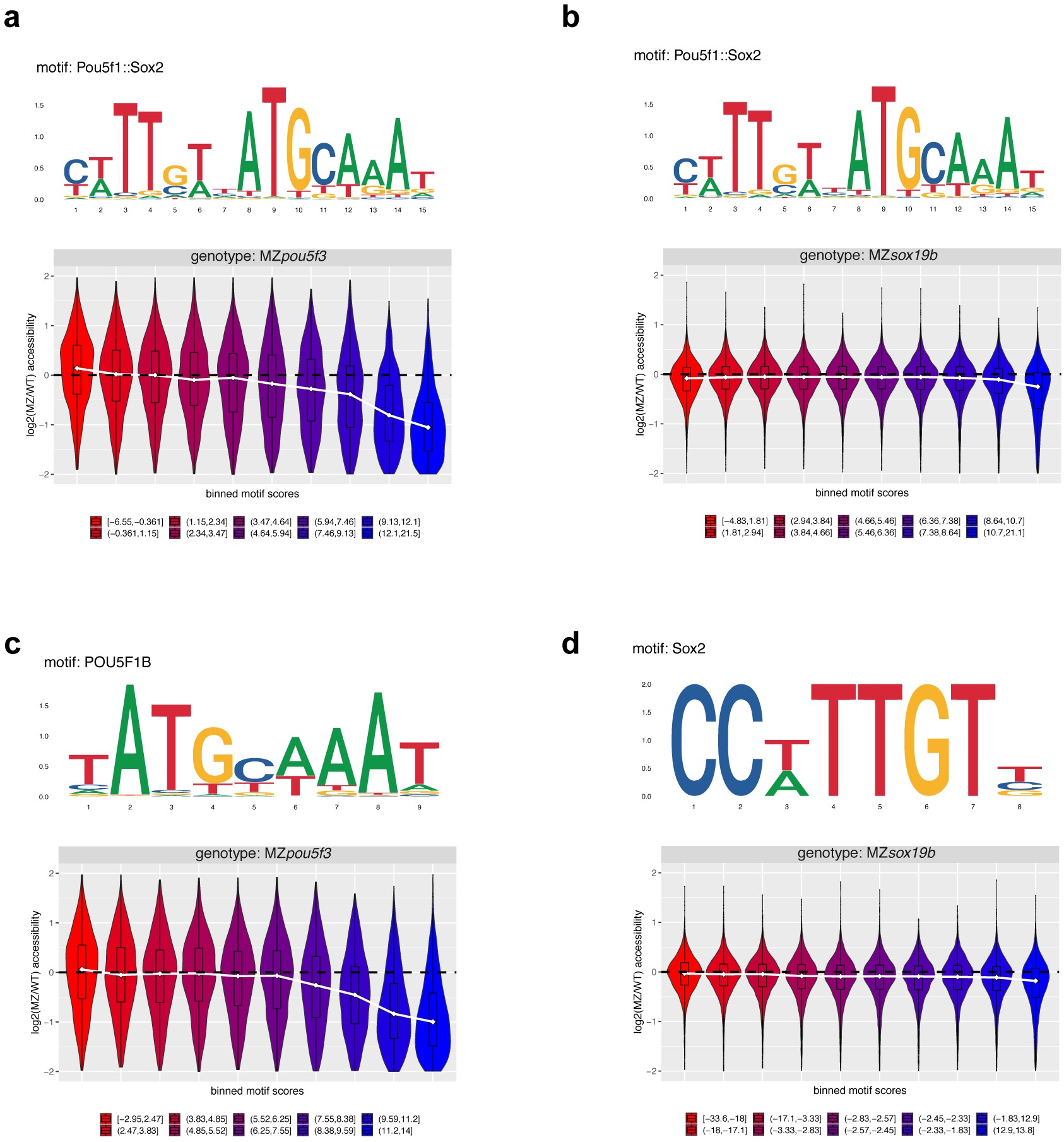

Supplement: S12 Fig — To determine the correlation between loss of accessibility in mutants and motif strength, we used motifs from the JASPAR 2018 database to perform low-stringency motif scanning. Binned motif scores are visualized from low (left) to high (right). Violin plots show the distribution of associated fold-changes in accessibility between a) MZpou5f3 and wild-type for the Pou5f1-Sox2 double motif; b) MZsox19b and wild-type for the Pou5f1-Sox2 double motif; c) MZpou5f3 and wild-type for the Pou5FB1 motif; and d) MZsox19b and wild-type for the Sox2 motif. (TIF) [file pgen.1008546.s012.tif]

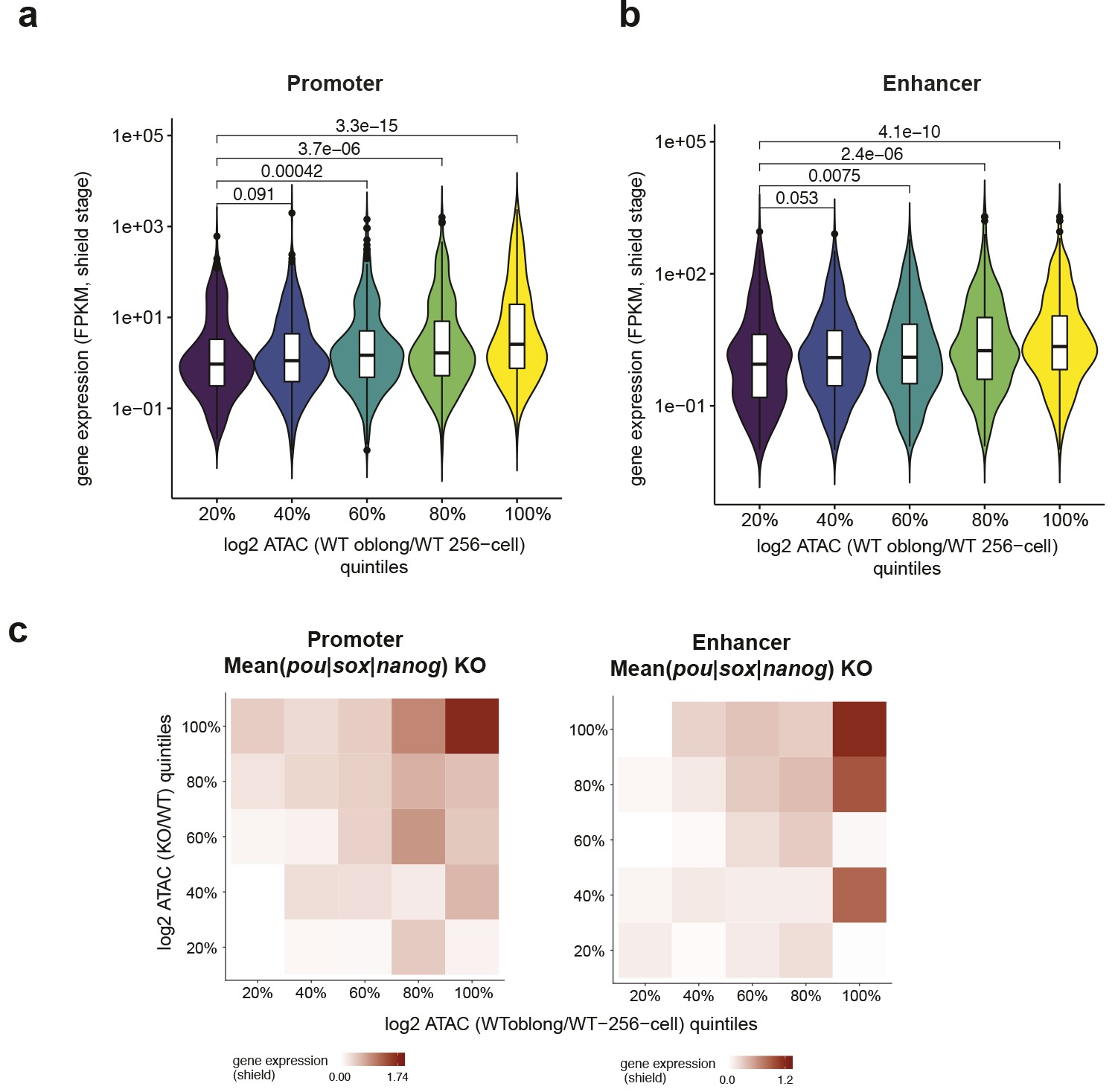

Supplement: S13 Fig — a) Promoter regions were sorted into 20% quintiles based on accessibility increase between 256-cell and oblong stage, and violin plots show the expression value of associated genes at shield stage. p-values are shown for differences in expression between quintiles as assessed by one-sided Wilcoxon tests. b) Putative enhancer regions were sorted into 20% quintiles based on accessibility increase between 256-cell and oblong stage, and violin plots show the expression value of associated genes at shield stage. p-values are shown for differences in expression between quintiles as assessed by one-sided Wilcoxon tests c) Heatmaps show the median expression value for genes associated with regulatory regions at shield stage. Genomic regions are resolved by 20% bins of accessibility increase between 256-cell and oblong stage (x-axis), and 20% bins of accessibility change in MZpou5f3, MZsox19b and MZnanog mutants compared to wild-type embryos at oblong stage (y-axis). (TIF) [file pgen.1008546.s013.tif]
