# Supplementary material for: Chromatin accessibility established by Pou5f3, Sox19b and Nanog primes genes for activity during zebrafish genome activation
Source: PLoS Genet. 2020 Jan 15;16(1):e1008546. doi: 10.1371/journal.pgen.1008546 (PMC6986763; doi:10.1371/journal.pgen.1008546)
Supplement: Attachment — Submitted filename: Palfy et al. Response_to_Reviewers.docx [file pgen.1008546.s014.docx]

**RESPONSE TO REVIEWERS’ SUGGESTIONS**

We thank the reviewers for their very thoughtful and supportive comments. In particular, the reviewers state that “the present manuscript complements and extends previous findings” and that “the study is sound and well-controlled”. They also write that “this is a very interesting and well written paper that reports convincing data to advance our understanding of the regulation of gene expression during development.”

Below, we address the reviewers’ comments point-by-point, using blue for the reviewers' comments and black for our responses. In the manuscript, we have highlighted text in yellow where it addresses specific points of the reviewers.

**Reviewers’ comments**

**Reviewer 1**

Zygotic genome activation is important for early embryonic development and the mechanisms underpinning this process should be of interest to the readership of PloS Genetics. Here Palfy and colleagues investigate ZGA in zebrafish, specifically focussing on the interplay between chromatin accessibility and transcription. They performed ATACseq on a series of early stages pre and post ZGA and found that from genome activation to onset of lineage specification chromatin accessibility increased in regulatory regions and preceded the transcription of associated genes. Using CRISPR mutants they show that this was dependent on the maternally expressed TFs, Pou5f3, Sox19B and Nanog, whose association with accessible elements was indicative of future transcription. They conclude that these TFs are involved in priming genes for transcription.

A lot is known about the timing of zygotic genome activation and Pou5f3, Sox19B and Nanog have previously been identified as key TFs involved. It was shown that they bind putative regulatory elements and a recent paper (Veil et al., Genome Research 2019) used MNase-seq and reports that these factors promote the open chromatin state. This study also provided chromatin accessibility data from similar stages (pre ZGA to dome stage). Thus, the present manuscript complements these previous reports and extends previous findings.

The experiments and associated bioinformatics analyses are well done, however, the overall advance is somewhat incremental.

They first attempt to address whether transcription is required for chromatin accessibility and compare a-amanitin treated and untreated embryos at the oblong stage, this treatment leads to arrest at sphere stage. Accessibility changes significantly only at the highly transcribed miR430 locus and not at any other highly transcribed genes. Thus, they conclude that RNA polymerase II activity is not required for chromatin accessibility, with the exception of the mir-430 cluster. It is not clear why they chose the id1 and fbxw4 loci to show ATACseq tracks and maybe the authors can comment on this selection also in the text (they do comment in the figure legend).

We apologize for this unclarity. The genes were chosen as representative examples for genes with strong zygotic expression and weak zygotic expression, respectively. This is now indicated in the text (p7) as well as the legend of Figure 2.

Looking at the timeline of developmental stages and publicly available expression data the authors observe that chromatin accessibility precedes transcription. They look at promoters and putative enhancers of genes that are activated at sphere and shield stages. The enhancers are defined by distance from TSS and presence of particular TF binding sites, but none of these putative enhancers is confirmed experimentally and their function and dependency on particular TFs is not confirmed.

We agree with the reviewer that enhancer predictions are not always correct. For this reason, we are showing data for enhancers that have been experimentally verified before in Figures 3 and S5. Moreover, the genes shown in Figure 3 and S5 that are expressed by shield stage (*olig4*, *gata6*, *ntla*) have been shown previously to be affected in Pou5f3 or Nanog knockdown at this stage (Lee et al., 2013). Please note that *neurog1* and *otx2* are expressed only at later stages, for which there is no expression data in the same paper.

They investigate the roles of Pou5f3, Sox19B and Nanog by creating CRISPR mutants followed by ATACseq at the oblong stage. The effects on accessibility at promoters and putative enhancers are quite small based on the volcano plots shown in Fig 4b, particularly in the MZsox19b and MZnanog mutants. Although for the selected genes shown in Fig 4c the effects are very clear and these genes and associated enhancers would be good candidates for experimental validation.

We agree with the reviewer that it is hard to see the extent of the effects in the volcano plots (Figure 4a). To facilitate the interpretation of the data, we have generated a boxplot (Figure S8) that compares accessibility between mutants and wildtype embryos. Statistical testing shows a clear effect on accessibility in all mutants.

In addition, we have added the wild-type data to the violin plots in Figure 4b to allow for statistical analysis. Here too we find significant changes at promoters and enhancers when comparing mutant with wildtype embryos.

Overall, their analysis confirms a previous publication which suggested that these TFs regulate chromatin accessibility. In addition, the authors suggest that sites bound by a single TF or two of the TFs have reduced accessibility in the mutants, but this is not very clear from the plots shown in Fig 4d.

We agree with the reviewer that the effects of mutants on sites that are bound by one or two TF are weaker than the effect on sites bound by three TF, but our analysis shows that they are statistically significant.

Finally, the authors find a correlation between increased accessibility of chromatin prior to gene expression at a subsequent stage and they suggest that Pou5f3, Sox19B and Nanog are priming gene expression.

The analysis reveals interesting associations, however the manuscript would benefit from experimental validations of some of the putative enhancers identified, including the confirmation of TF binding sites as being functionally important.

As indicated above, some of the enhancers have been experimentally verified before. Although it would be interesting to test more of them, we feel that this is beyond the scope of this study.

**Reviewer 2**

This manuscript investigates the process of zygotic genome activation (ZGA) in zebrafish embryos to explore the relationship between transcription, chromatin accessibility and how this is regulated, including the regulatory proteins involved. These are interesting and important questions because we still do not fully understand how chromatin accessibility is regulated and how changes in accessibility are related to gene expression during development i.e. whether open chromatin is a reliable predictor the genes being expressed in cells or the future genes that will be expressed.

The authors carry out ATAC-Seq in replicates at seven different stages of zebrafish embryogenesis covering from the 256 cell stage - approximately when zygotic gene expression begins – to gastrulation. I was curious however as to why earlier stages were not assayed since the authors themselves state that some zygotic transcription is observed earlier at the 64-cell stage.

We agree with the reviewer that it would be interesting to analyze earlier developmental stages given that some transcriptional activity is detected as early as 64-cell stage. However, we aimed to compare accessible chromatin to future transcriptional activity and have chosen to analyze stages that would give us a significant number of peaks to work with. Because even at 256-cell stage, a relatively low number of peaks was detected, we decided not to analyze earlier stages.

Comparison of the ATAC-Seq profiles across these stages allowed the authors to visualise and compare changes in open chromatin and how this is associated with changes in gene expression. They found five clusters of genes with different chromatin accessibility dynamics representing increasing numbers of genes with promoters and enhancers that become more accessible as development proceeds. They then provide convincing evidence that this increasing accessibility is generally not dependent on transcription except for the very highly expressed miR-430. They then show that accessibility of promoters and enhancers preceeds the expression of the associated genes. This is an important result that, consistent with data from other organisms, strongly suggests while generally open chromatin is not a great predictor of which genes are expressed at a given stage or tissue, it can be predictive of future gene expression during development.

The authors then investigate the effect of loss of three candidate transcription factors on chromatin accessibility during ZGA: Pou5f3, Sox19b and Nanog. They found that loss of any of these factors results in a decrease in chromatin accessibility and that this effect is caused by a loss of binding of these factors individually and in combination at different loci. Analysis of gene expression evidences that these three transcription factors likely prime genes for expression by increasing chromatin accessibility.

Overall I think this is a very interesting and well written paper that reports convincing data to advance our understanding of the regulation of gene expression during development.

We thank the reviewer for his/her enthusiasm.

**Reviewer 3**

The paper by Palfy and colleagues explores the potential roles of maternal genes Pouf5f3, Sox19b and Nanog as “pioneer factors” that help open the chromatin and prepare for zygotic transcription. This study is sound and well-controlled, and although much work has already been done in exploring chromatin accessibility profiles before gene transcription as well as ChIP of the studied factors, this study provides critical mutant comparisons as well as new KO data for all three factors including Sox19b. The study is very well executed, and the manuscript was written clearly; however, data is insufficiently explored. A few suggested experiments, additional analyses and change in terminology would help make the study stronger and would help support better the claims the authors make.

In short, the description of downstream genes affected in mutants is lacking; where do the factors act to open the chromatin? Is this correlated with gene expression? RNA-pol II activity cannot be used as a single readout of gene expression. RNA-seq heatmap at the stages analysed and correlation of the ATAC-seq, RNA-seq and previously published ChIP-seq would greatly enhance the author’s claims. Importantly, further mining of the ATAC-seq data in mutant conditions, in combination with previously published ChIP data at the “pioneering stages” will be essential to identify the imprint of the pioneering factors on the chromatin.It has been shown recently that transcription factors only recognise partial DNA binding motifs when pioneering (Yan, Chen, & Bai, Mol Cell (2018); Mayran & Drouin, JBC (2018); Lukeseviciute et al., Dev Cell(2018); Meers, Janssens& Henikoff, Mol Cell (2019)). By analysing only canonical TF binding (known motifs), the authors may not be recognising the pioneering/priming per se but actual enhancing/co-operative activity required for transcriptional gene activation. Identifying TF factor-bound regions (existing ChIP data) that have defective “opening” in mutants and running random searches for de novo motifs will provide insight into new biology not accessed before, warranting much higher impact.

We thank the reviewer for these suggestions. We address them below.

The question whether the opening of chromatin by TFs is correlated with gene expression is partly addressed in Figure 5a-d where we show that accessibility, especially at regions opened by Pou5f3, Sox19b and Nanog, correlates with future gene expression and future H3K27Ac marking. We apologize that this was unclear. We have revised the text to emphasize this point (p12, 13).

In addition, we now determined where the factors act to open chromatin, and whether this is correlated with gene expression. To this end, we compared the effects of loss of Pou5f3/Sox19b/Nanog on accessibility in our mutants with the effects of Pou5f3/SoxB1/Nanog on gene expression that have been reported before (Lee et al., 2013). As can be observed in the new Figure S9 , this analysis confirms that changes in accessibility of promoters and enhancers are significantly larger for genes whose expression is affected in mutants than for genes for which no such gene expression change has been reported. A sentence to report this has been added (p10).

Following the suggestion of the reviewer to look into the importance of partial motifs, we performed two kinds of analysis. First, to remove any bias in our search, we did a de novo motif search on the sites that are most affected in accessibility in mutants compared to wt. For all three TFs we allowed for multiple motifs of varying lengths to be discovered, yet significantly enriched motifs only showed minimal deviations from the respective canonical binding sites (Figure S11). This result suggests that motif strength is a good predictor for the observed difference in accessibility between wt and mutant embryos. To further analyze this, we then took the canonical binding motifs for Pou5f3, Sox19b and Nanong and made 10 bins of increasing motif score, with the highest score (on the right) representing the canonical binding motif. This indeed revealed that the largest change in accessibility is observed for sites with the canonical binding site (Figure S12). Taken together, this suggests that in zebrafish, Pou5f3, Sox19b or Nanog do not preferentially bind partial motifs at sites where they open up chromatin, in contrast to what has been reported during stem cell reprogramming (Soufi et al. 2015). A recent study came to a similar conclusion (see Veil et al. 2019, Figure 6). A paragraph has been added to describe these results (p11).

Detailed comments are listed below:

Figure 1 – Notion of gradual accessibility should be corrected. There is no such thing as “gradual” opening, and the read counts reflect a number of cells (proportions of cells if normalised) that harbour a given accessible element.

We thank the reviewer for this comment as it has forced us to be more precise. We agree that an increase in accessibility at a given region can be due to more cells in which the region is open. However, given that most cells are the same at the developmental stage at which we performed our analysis, we think that heterogeneity cannot be the primary cause for the increase we observe (also see discussion). Instead, because genomic regions are found in a continuum of accessibility states (Klemm et al. 2019), our interpretation of the data is that regulatory elements transition to a higher accessibility state during genome activation. This will increase the probability that a given regulatory element is found open in a cell. To better reflect this thinking, we have changed the wording throughout the manuscript and in the discussion.

Figure 2 – Please provide a reference supporting the claim that an increase in chromatin accessibility during genome activation suggests that accessibility could be a consequence of transcriptional activity? Moreover, I am not sure what Figure 2 brings to the paper since the next section immediately disproves the claims. An addition of RNA-seq data, correlation and biological interpretation will help support the claims in Figures 1 and 2.

We apologize for the confusion here. We previously wrote “The increase in chromatin accessibility during genome activation suggests that accessibility could be a consequence of transcriptional activity”. No reference is given because it is our own data in Figure 1 that suggests this. We have now added “that we observe” to the sentence above (p6) to emphasize that this hypothesis is based on our own data.

We investigate this possibility in Figure 2 and find that, in fact, the increase in chromatin accessibility is generally *not* a consequence of transcriptional activity (with the one exception being the hypertranscribed miR-430 cluster). This is in line with the data in Figure 3 that shows that the increase in accessibility precedes transcriptional activity.

Figure 3 – It is already known that chromatin is prepared before transcription and needs to be in a poised state before transcription happens. While it is good to have the confirmation once more, given the absence of novelty and a lack of impact, maybe this figure could be combined with Figure 1.

Here we respectfully disagree with the reviewer. Although there is indeed work that shows that chromatin is accessible prior to transcription, it is often assumed that accessibility can be used as a proxy for transcriptional activity. Thus, it is not so clear that accessibility precedes transcription. Above, reviewer 1 writes “They then show that accessibility of promoters and enhancers precedes the expression of the associated genes. This is an important result that, consistent with data from other organisms, strongly suggests while generally open chromatin is not a great predictor of which genes are expressed at a given stage or tissue, it can be predictive of future gene expression during development.” emphasizing the importance of this analysis.

Figure 4 – Please provide more information on why the oblong stage was chosen for comparison here?

Oblong stage was chosen because it is the last developmental stage during which development still relies entirely on maternal products (transcription-inhibited embryos developmentally arrest one stage later, at sphere stage). This makes it unlikely that the effects on accessibility we see in the mutants are indirect (for example, when loss of the transcription factor would result in reduced expression of another chromatin opening factor). We have clarified this in the text (p10).

Figure 4b – Statistics associated with the claims should be presented. Were the observed differences statistically significant?

We thank the reviewer for pointing this out. We have now compared the accessibility of promoter and enhancer regions between mutant and wild-type conditions (paired t-tests) and found a significantly lower average accessibility in mutant conditions.

Figure 5 – Before concluding, the authors need to clarify whether the overexpression results in the opening of more chromatin areas to consolidate this finding that these are factors essential for this activity, i.e. whether they act as pioneer factors? Correlation with the previous chromatin binding maps should be included.

We thank the reviewer for this suggestion. We do not expect that overexpression of Pou5f3, Sox19b or Nanog in the early embryo would result in many more open sites, because these factors are already present at high levels (in fact, they are the most highly expressed transcription factors in the early zebrafish embryo - Lee et al. 2013). Thus, it is hard to imagine that further increasing their levels would have a large effect. In fact, in a recent study the overexpression of Nanog, SoxB1, or Pou5f3 did not result in premature genome activation in zebrafish (Chan et al. 2019). However, the importance of the three TFs in opening chromatin is shown by the observation that accessibility is lost in knock-outs.

Further comments:

1. Terminology on the dynamic increase in chromatin accessibility: The increase in chromatin accessibility is due to a greater number of cells with opened chromatin (evicted nucleosomes) at a given locus rather than “progressive/partial” accessibility. The cell either has a particular locus accessible or not.

Please see our answer above.

2. Better referencing to support the hypotheses and discuss the claims.

As suggested by the reviewer, we have added additional references in the part where we discuss whether partial motifs play a role in binding of pioneer factors (Mayran & Drouin, JBC (2018), Meers, Janssens & Henikoff, Mol Cell (2019). In addition, we added references pertaining to the interpretation of increasing accessibility (Klemm, Shipony & Greenleaf, Nat Rev Genetics (2019), Buenrostro et al, Nature (2015), Cusanovich et al, Science (2015)).

3. Statistical tests should be presented for the mutant vs control analyses.

We have now added statistics to Figure 4b and have performed and added additional analyses with statistical tests to look at the effect on accessibility between mutants and controls (Figures S8, S9).
